# Antioxidant Effect of Barley Sprout Extract via Enhancement of Nuclear Factor-Erythroid 2 Related Factor 2 Activity and Glutathione Synthesis

**DOI:** 10.3390/nu9111252

**Published:** 2017-11-16

**Authors:** Yun-Hee Lee, Sou Hyun Kim, Seunghyun Lee, Kyung-Mi Kim, Jae-Chul Jung, Tae Gen Son, Sung Hwan Ki, Woo-Duck Seo, Jae-Hwan Kwak, Jin Tae Hong, Young-Suk Jung

**Affiliations:** 1 College of Pharmacy, Yonsei University, Incheon 21983, Korea; yunhee.lee@yonsei.ac.kr; 2 College of Pharmacy, Pusan National University, Busan 46241, Korea; hyunie9808@naver.com (S.H.K.); tmdgus4374@naver.com (S.L.); 3 Life Science Research Institute, Novarex Co., Ltd., Ochang, Cheongju 28126, Korea; kkm3507@novarex.co.kr (K.-M.K.); jcjung@novarex.co.kr (J.-C.J.); 4 Division for Research Center, Dongnam Institute of Radiological and Medical Science, Busan 46033, Korea; tgson@hanmail.net; 5 College of Pharmacy, Chosun University, Gwangju 61452, Korea; shki@chosun.ac.kr; 6 National Institute of Crop Science(NICS), Rural Development Administration(RDA), Jeollabuk-do 55365, Korea; swd2002@korea.kr; 7 College of Pharmacy, Kyungsung University, Busan 48434, Korea; jhkwak@ks.ac.kr; 8 College of Pharmacy, Chungbuk National University, Cheongju 28160, Korea; jinthong@chungbuk.ac.kr

**Keywords:** barley sprouts, alcohol-induced liver injury, oxidative stress, Nrf2, glutathione

## Abstract

We previously showed that barley sprout extract (BSE) prevents chronic alcohol intake-induced liver injury in mice. BSE notably inhibited glutathione (GSH) depletion and increased inflammatory responses, revealing its mechanism of preventing alcohol-induced liver injury. In the present study we investigated whether the antioxidant effect of BSE involves enhancing nuclear factor-erythroid 2 related factor 2 (Nrf2) activity and GSH synthesis to inhibit alcohol-induced oxidative liver injury. Mice fed alcohol for four weeks exhibited significantly increased oxidative stress, evidenced by increased malondialdehyde (MDA) level and 4-hydroxynonenal (4-HNE) immunostaining in the liver, whereas treatment with BSE (100 mg/kg) prevented these effects. Similarly, exposure to BSE (0.1–1 mg/mL) significantly reduced oxidative cell death induced by *t*-butyl hydroperoxide (*t*-BHP, 300 μM) and stabilized the mitochondrial membrane potential (∆ψ). BSE dose-dependently increased the activity of Nrf2, a potential transcriptional regulator of antioxidant genes, in HepG2 cells. Therefore, increased expression of its target genes, heme oxygenase-1 (HO-1), NADPH quinone oxidoreductase 1 (NQO1), and glutamate-cysteine ligase catalytic subunit (GCLC) was observed. Since GCLC is involved in the rate-limiting step of GSH synthesis, BSE increased the GSH level and decreased both cysteine dioxygenase (CDO) expression and taurine level. Because cysteine is a substrate for both taurine and GSH synthesis, a decrease in CDO expression would further contribute to increased cysteine availability for GSH synthesis. In conclusion, BSE protected the liver cells from oxidative stress by activating Nrf2 and increasing GSH synthesis.

## 1. Introduction

Chronic alcohol consumption results in alcoholic liver disease (ALD) that encompasses a spectrum of injury, ranging from fatty liver disease to irreversible cirrhosis [1]. ALD is one of the major causes of morbidity and mortality worldwide, and multiple factors, including sex, age, and ethnicity, influence the disease progression [2]. Alcohol induces oxidative stress by free radical formation, which mainly contributes to liver injury [3]. Ethanol is oxidized to toxic metabolite, acetaldehyde. Cytochrome P450 2E1 (CYP2E1) in the hepatocytes is mainly responsible for the metabolism of ethanol and these toxic metabolites induce NADPH oxidase activity in the Kupffer cells (liver-resident macrophages), abnormal mitochondrial function, disturbance in lipid metabolism, and cytokine production [4,5,6]. Chronic alcohol consumption induces hepatic CYP2E1, which not only accelerates oxidation of ethanol to acetaldehyde, but also generates reactive oxygen species (ROS) [7]. Transgenic mice overexpressing CYP2E1 exhibit enhanced oxidative stress and exacerbated alcohol-induced liver injury [8,9]. In contrast, deletion of CYP2E1 protects mice from chronic alcohol consumption-induced liver injury via reduction of ROS [8].

Impaired hepatic antioxidant enzymes and disturbed hepatic glutathione (GSH) homeostasis further contribute to the damage by oxidative stress and associated alcoholic liver injury. A decrease in hepatic glutathione (GSH) is one of the early changes associated with chronic alcohol consumption that drives ALD pathogenesis [10]. Chronic alcohol supplementation in baboons significantly depleted hepatic GSH along with its precursor S-adenosylmethionine (SAM); recovery of GSH level was affected by SAM administration [11]. In an acute model of early-stage ALD, SAM treatment prevented the depletion of hepatic GSH and increase in lipid peroxidation, thereby attenuating alcohol-induced liver injury [12]. Preclinical studies verify that N-acetylcysteine, another precursor of GSH, prevents oxidative liver injury after chronic alcohol exposure [13,14]. Therefore, restoration of the decreased hepatic GSH and minimizing alcohol-induced oxidative stress could be an effective strategy to prevent ALD.

Nuclear factor-erythroid 2 related factor 2 (Nrf2) is a transcription factor regulating the expression of antioxidant genes and other cyto-protective enzymes [15]. Target genes of Nrf2 include NADPH quinone oxidoreductase 1 (NQO1), glutamate-cysteine ligase catalytic (GCLC) and modifier (GCLM) subunit, and many other protein repair genes [15,16]. Under normal conditions, Nrf2 is sequestered by kelch-like ECH associated protein 1 (Keap1) in the cytosol; however, it is released from Keap1 in response to oxidative stress and translocated into the nucleus, where it triggers cyto-protective and cyto-adaptive responses [15,17]. Nrf2 knockout potentiates hepatotoxicity induced by acetaminophen, alcohol, and methionine-choline deficient diet through increased oxidative stress [18,19,20]. However, pharmacologic or genetic induction of Nrf2 activity effectively prevents oxidative stress-induced tissue injury [20,21,22]. Therefore, current antioxidant therapies focus on Nrf2 activation by natural or synthetic compounds.

Young leaves of barley (*Hordeum vulgare* L.) or barley sprouts, harvested 10 days after sowing, contain various ingredients that are beneficial to health. We previously demonstrated that barley sprout extract (BSE) prevents liver injury induced by chronic alcohol intake in mice by suppressing inflammatory responses and preventing GSH depletion [23]. Specifically, BSE prevents LPS-stimulated expression of nitric oxide synthase and cyclooxygenase-2 in RAW 264.7 cells, which reveals its mechanism of alleviation of alcoholic liver injury. In this study, we evaluated whether BSE prevents oxidative stress in the livers of mice treated with alcohol, and promotes GSH synthesis through Nrf2 activation to overcome alcohol-induced oxidative stress.

## 2. Materials and Methods 

### 2.1. Preparation of BSE

We obtained barley sprouts cultivated in Yeonggwang-gun, Jeollanam-do Province, Korea from Saeddeumwon Co., Ltd. (Yeonggwang, Korea) in 2015 and the extract was produced by Novarex Co., Ltd. (Ochang, Korea). Briefly, the sprouts were exposed to circulating aqueous fermented ethanol (30%) for 9 h at room temperature. After filtration and centrifugation, the supernatant was concentrated to attain 35 brix, and BSE in powder form was obtained by sterilization and spray-drying.

The extraction efficiency was calculated based on the content of saponarin, a major compound in barley sprouts, by high-performance liquid chromatography (HPLC) with a UV detector (Agilent Technologies 1260 Infinity, Palo Alto, CA, USA). Chromatographic separation was performed on a reversed-phase HPLC column (Capcell Pak C18, 4.6 mm × 250 mm; Shiseido, Tokyo, Japan) at 40 °C, with the mobile phase as 0.1% formic acid in water (A) and 0.1% formic acid in acetonitrile (B) at a flow rate of 1 mL/min. The gradient program was as follows: 0 min, 10% B; 0–32 min, 10–40% B; 32–35 min, 40–60% B; 35–42 min, 60–10% B. The injection volume was 10 μL and the detection wavelength was 260 nm.

### 2.2. Animal Experiments

Male C57BL/6NKorl mice were procured from the Department of Laboratory Animal Resources at the National Institute of Food and Drug Safety Evaluation (NIFDS, Cheongju, Korea). Animals were acclimated to temperature (22 ± 2 °C) and humidity (55 ± 5%) controlled rooms with a 12-h light/dark cycle for 1 week prior to initiation of the experiment. Mice were fed a Lieber–DeCarli liquid diet (Dyets Inc., Bethlehem, PA, USA) with or without alcohol for 4 weeks. The control diet was nutritionally composed of fat (35%), protein (18%), and carbohydrates (47%); the alcohol-supplemented diet contained fat (35%), protein (18%), carbohydrates (11%), and ethanol (36%). BSE was administered daily by gavage throughout the 4 weeks of alcohol treatment. The animal experiment protocol complied with the guidelines established and approved by the Animal Care and Use Committee in Pusan National University (Approval No. PNU-2015-1027).

### 2.3. Determination of Lipid Peroxidation in the Liver Tissue

Lipid peroxidation in the liver tissue was determined by the thiobarbituric acid reactive substances (TBARS) assay as described by Volpi and Tarugi [24]. The liver tissue was homogenized with 3 volumes of cold 1.15% KCl solution. The lysate was then incubated with 0.2% thiobarbituric acid in sodium acetate buffer (2 M, pH 3.5) containing 5% butylated hydroxytoluene in ethanol at 95 °C for 45 min. After centrifugation, the supernatant was injected into an HPLC system equipped with a fluorescence detector (FLD-3100; Thermo Scientific, Sunnyvale, CA, USA) and a 5 μm Symmetry C18 reversed-phase column (4.6 mm × 150 mm; Eka Chemicals, Bohus, Sweden). The mobile phase was composed of 35% methanol and 65% sodium phosphate buffer (50 mM, pH 7.0). The malondialdehyde-thiobarbituric acid complex was monitored by fluorescence detection (ex/em: 515/553 nm).

### 2.4. Immunohistochemical Staining of 4-Hydroxynonenal (4-HNE)

To evaluate hepatic 4-HNE adducts, a cross-section (5 µm) of the left lateral lobe of the liver was sliced and stained by rabbit polyclonal anti-4-HNE (Abcam, Cambridge, MA, USA) and goat anti-rabbit polyclonal antibodies (Vectastain ABC IHC kit, Vector Laboratories Inc., Burlingame, CA, USA).

### 2.5. Cell Culture

Human liver HepG2 cells were obtained from the American Type Culture Collection (Manassas, VA, USA) and grown in Dulbecco’s modified eagle’s medium (GenDEPOT, Barker, TX, USA) containing 10% fetal bovine serum, glutamine (2 mM), penicillin (100 U/mL), and streptomycin (100 μg/mL) at 37 °C in a humidified incubator with 5% CO_2_.

### 2.6. Measurement of ROS Generation 

ROS generation was determined with a fluorescent probe, dichlorofluorescein diacetate (DCFH–DA). HepG2 cells were pre-incubated with BSE for 3 h and then incubated with tert-butyl hydroperoxide (*t*-BHP) for another 1 h. The cells were stained with 10 DCFH–DA (μM) by incubating for further 1 h. The fluorescence intensity in the cells was measured using a Glomax Explorer System (Promega, Madison, WI, USA). ROS production was calculated relative to the vehicle-treated control.

### 2.7. Determination of Cell Viability

HepG2 cells were plated in a 96-well plate and cell viability was determined 18 h after *t*-BHP treatment by the MTT assay as instructed by the manufacturer. Briefly, after incubation with MTT (0.5 mg/mL) for 4 h at 37 °C, the formazan (precipitated by the action of mitochondrial dehydrogenases in viable cells) was extracted with dimethyl sulfoxide (DMSO). The absorbance of the converted dye was measured at 540 nm using a Multiskan GO reader (Thermo Scientific, Sunnyvale, CA, USA), and the results were expressed as a percentage of vehicle-treated viable cells.

### 2.8. Fluorescence-Activated Cell Sorting (FACS) Analysis of Apoptosis 

Both apoptotic and live cells were determined by Fluorescence-Activated Cell Sorting (FACS) analysis after annexin V-FITC staining. HepG2 cells were incubated with BSE for 12 h and later treated with *t*-BHP (300 μM) for 18 h. Subsequently, the cells were harvested, trypsinized, washed once in cold phosphate-buffered saline (PBS), and suspended in 1× binding buffer. The counted cells were stained with propidium iodide and annexin V-FITC (BD Biosciences, Bedford, MA, USA) at room temperature for 15 min in the dark. The stained cells were analyzed by flow cytometry within 1 h. Both apoptotic and live cells were analyzed by a BD FACScan flow cytometer and BD FACSDiva software (BD Biosciences, San Jose, CA, USA).

### 2.9. FACS Analysis of Mitochondrial Membrane Potential (∆ψ)

HepG2 cells were incubated with BSE for 12 h and later treated with *t*-BHP (300 μM) for 18 h. The cells were then incubated with JC-1 (10 μM) in the culture medium for 30 min in the dark and collected by scraping. After washing in PBS, the cells were subjected to FACS analysis. JC-1 requires 2 excitation wavelengths, 527 nm (green) for the monomer form and 590 nm (red) for the aggregate form. With normal mitochondrial function, the ∆ψ is high and the red fluorescence is predominant. However, mitochondrial injury reduces the ∆ψ, observed as an increase in green fluorescence. Quantitation of red and green fluorescence allows estimation of mitochondrial damage. The change in ∆ψ was monitored a BD FACScan flow cytometer and BD FACSDiva software (BD Biosciences, San Jose, CA, USA).

### 2.10. Antioxidant Response Element (ARE) Luciferase Assay

An NQO1-ARE firefly luciferase construct, containing 3 tandem repeats of the ARE in the 5′-upstream region of NQO1, was introduced into the cells to determine transcriptional activation of Nrf2 by BSE. HepG2 cells were plated overnight in 12-well plates, serum-starved for 6 h, and then transfected with the firefly luciferase construct and pRL-SV plasmid (*Renilla* luciferase) in the presence of Lipofectamine^®^ 2000 (Invitrogen, San Diego, CA, USA) for 5 h. The firefly luciferase activity was measured by adding the luciferase assay reagent II (Promega, Madison, WI, USA) according to the manufacturer’s instructions, and the *Renilla* luciferase activity was determined by adding Stop & Glo^®^ reagent (Promega, Madison, WI, USA). Relative luciferase activities were calculated by normalizing firefly luciferase activity with that of *Renilla* luciferase.

### 2.11. Real-Time Reverse Transcription-Polymerase Chain Reaction (RT-PCR)

The total RNA was isolated from cells using the RNeasy kit (Qiagen, Valencia, CA, USA). The cDNA was synthesized by the iScriptTM cDNA Synthesis system (Bio-Rad, Hercules, CA, USA). The real-time RT-PCR was performed by using the SensiFAST SYBR qPCR mix (Bioline, London, UK) according to the manufacturer’s protocol. The relative values of gene expression were normalized to Glyceraldehyde-3-phosphate dehydrogenase (GAPDH). The primer sequences are provided in Table 1.

### 2.12. Western Blotting

Cells were lysed with ice-cold PRO-PREP™ protein extract solution (iNtRON, Sungnam, Gyunggi, Korea) and the protein concentration was determined by the BCA assay (Thermo Scientific). Equal amounts of protein were separated by SDS-PAGE and then transferred onto a polyvinylidene difluoride membrane (Millipore, Billerica, MA, USA). The membrane was blocked with 5% skim milk in Tris-HCl (100 mM, pH 7.5), NaCl (150 mM), and 0.2% Tween-20 (TBST) for 1 h at room temperature. The membranes were incubated with TBST containing 5% skim milk and primary antibodies against Nrf2, transcription factor II B (TFIIB), NQO1, GAPDH, and α-tubulin (Santa Cruz Biotechnology, Santa Cruz, CA, USA); heme oxygenase (HO-1, Enzo Life Sciences, Farmingdale, NY, USA); cysteine dioxygenase (CDO) and GCLC (Abcam). After washing with TBST, the blot was incubated with the appropriate horseradish peroxidase (HRP)-conjugated secondary antibodies and visualized using an enhanced chemiluminescent HRP substrate kit (Western Bright, Advansta, Menlo Park, CA, USA).

### 2.13. Determination of Cysteine, Taurine, and GSH Levels

HepG2 cells were plated in 100-mm culture plates for 24 h and were harvested after a medium change by scraping in either 5% perchloric acid to determine the cysteine and GSH level, or in ice-cold methanol to determine the taurine level. The protein pellet was dissolved in NaOH (0.1 M) solution, and the protein concentration was determined using a BCA protein assay kit (Thermo Scientific). Liver tissues were homogenized in a 4-fold volume of 1 M perchloric acid to determine cysteine level. Denatured protein was removed by centrifugation. Taurine was derivatized with *ο*-phthalaldehyde/2-mercaptoethanol and quantified using HPLC with a fluorescence detector (FLD-3100; Thermo Scientific; ex/em: 338/425 nm); a Hector T-C18 column (3 μm × 4.6 mm × 100 mm) was used [25,26]. Cysteine and GSH was quantified using the 7-fluorobenzofurazan-4-sulfonic acid ammonium salt (SBD-F) pre-column derivatization method [27]. MPG (50 μL), as an internal standard, was added to each sample (50 μL) and briefly vortex-mixed. Following addition of 10 μL of a 10% (*w*/*v*) tris(2-carboxyethyl)phosphine solution, the samples were incubated at room temperature for 30 min. Subsequently, 90 μL of 10% (*w*/*v*) trichloroacetic acid solution with EDTA (1 mM) was added to each sample, and the samples were briefly vortex-mixed and centrifuged (13,000× *g*, 10 min). The supernatants (50 μL) were then mixed with 10 μL of NaOH (1.55 M), 125 μL of borate buffer (0.125 M, pH 9.5) with EDTA (4 mM), and 50 μL of 0.1% (*w*/*v*) SBD-F in borate buffer (0.125 M) with EDTA (4 mM). After incubating at 60 °C for 1 h, 20-μL aliquots were injected into an HPLC system equipped with a fluorescence detector (ex/em: 385/515 nm); chromatographic separation was achieved using a Hector M-C18 column (3 μm × 4.6 mm × 150 mm).

### 2.14. Statistical Analysis

All results, expressed as mean ± SD, were analyzed by one-way analysis of variance (ANOVA) followed by Newman–Keuls multiple comparisons test or unpaired Student’s *t*-test. The acceptable level of significance was established at *p* < 0.05.

## 3. Results

### 3.1. Standardization of BSE 

**Figure 1:**
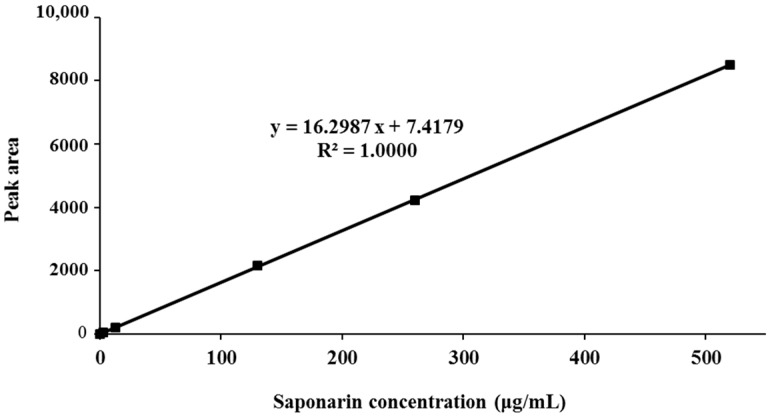
Calibration curve for saponarin standard. y, peak area (mAU) of saponarin; x, concentration (μg/mL) of saponarin; *R*^2^, correlation coefficient.

**Figure 2:**
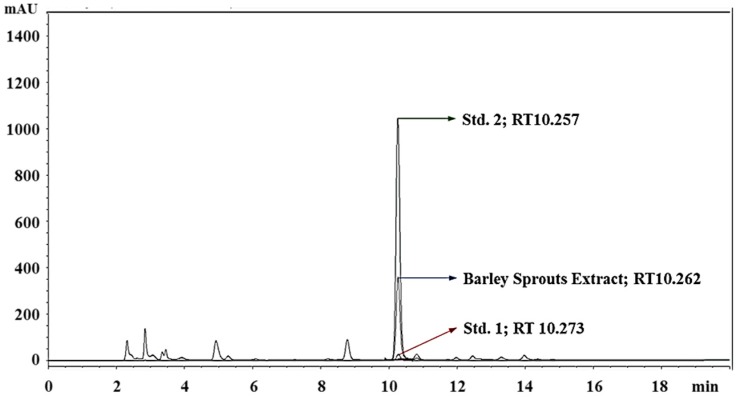
Standardization of barley sprout extract by determination of saponarin by HPLC. Std. 1, saponarin (1 µM); Std. 2, saponarin (10 µM); RT, retention time.

Saponarin, a flavone glucoside, is the major ingredient of barley sprouts. The BSE was standardized based on the HPLC quantification of saponarin. Linearity of the HPLC method was assessed by injecting five concentrations of a standard saponarin solution. The calibration curve showed good linearity (*R*^2^ = 1) in the range of 0–520 μg/mL (Figure 1). Saponarin in the extract was identified and quantified (14.74 ± 0.27 μg/mg) by comparing the peak retention time and area with those of standard saponarin (Figure 2).

### 3.2. Prevention of Alcohol-Induced Oxidative Liver Injury by BSE

**Figure 3:**
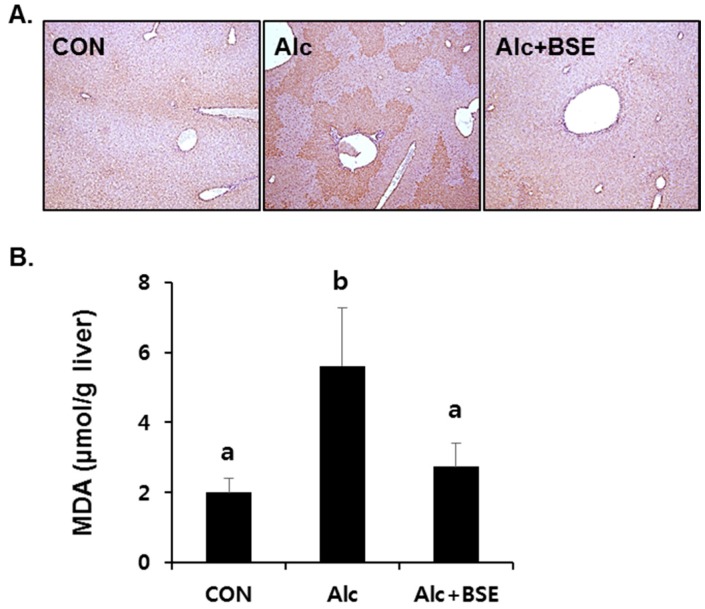
Lipid peroxidation in the livers of alcohol-fed mice with or without barley sprout extract (BSE) for 4 weeks. (**A**) Immunohistochemistry of 4-HNE in the liver tissue and (**B**) malondialdehyde (MDA) level in the liver homogenate. Each value is presented as mean ± SD of 6 mice. Values with different letters are significantly different by analysis of variance (ANOVA) followed by Newman–Keuls multiple comparisons test (*p* < 0.05). CON, control diet-fed mice; Alc, alcoholic diet-fed mice; Alc+BSE, alcoholic diet-fed mice treated with barley sprout extract (100 mg/kg).

Our previous results showed that BSE protects against liver injury induced by chronic alcohol ingestion [23]. To determine whether BSE prevents oxidative stress in alcoholic liver injury, the extent of lipid peroxidation was determined in the liver tissues of mice by 4-HNE immunostaining and the TBARS assay. 4-HNE immunopositivity was clearly observed in the fixed liver tissues of alcohol-fed mice for four weeks, which was absent in the liver tissues of mice treated together with alcohol and 100 mg/kg of BSE (Figure 3A). Similarly, the alcohol insult significantly increased the concentration of MDA–TBARS in the liver homogenates, whereas supplementation of BSE in alcohol-fed mice prevented this increase (Figure 3B).

### 3.3. Inhibition of t-BHP-Induced Oxidative Cell Death by BSE in HepG2 Cells

**Figure 4:**
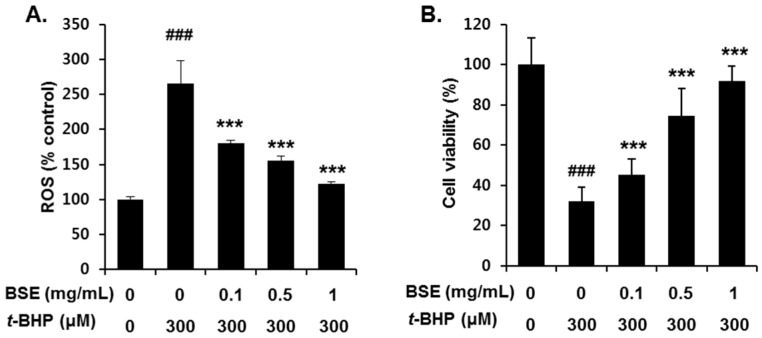
Inhibition of *t*-BHP-induced (**A**) ROS generation and (**B**) cell viability by barley sprout extract (BSE) in HepG2 cells. Cells were pretreated with BSE ranging from 0.1 mg/mL to 1 mg/mL for 3 h before treatment with *t*-BHP (300 µM) for 1 h to measure ROS generation. To determine cell viability, the cells were pretreated with BSE for 12 h before treatment with *t*-BHP (300 µM) for 18 h. Each value represents the mean ± SD. ^###^ Significantly different from vehicle-treated cells, *p* < 0.001. *** Significantly different from *t*-BHP-treated cells, *p* < 0.001 (Student’s *t*-test).

**Figure 5:**
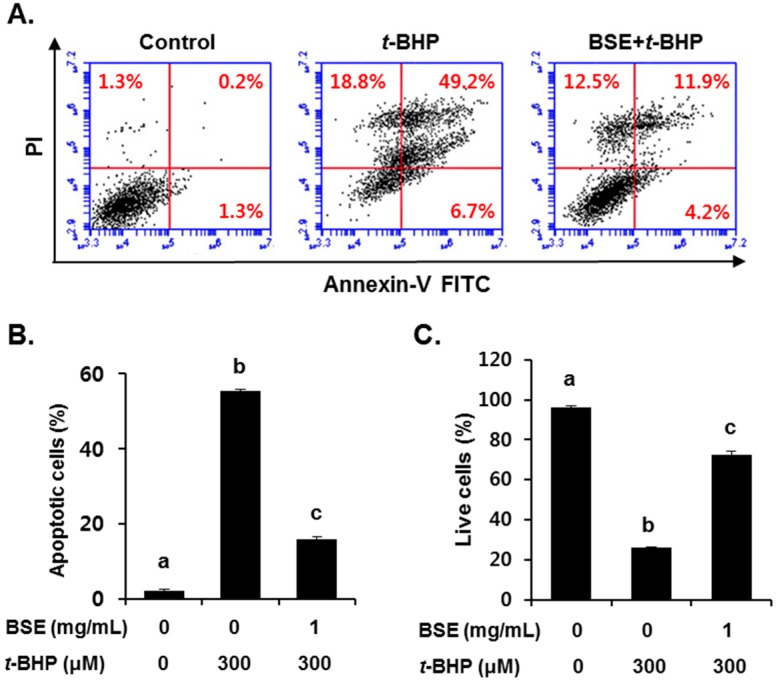
Inhibition of *t*-BHP-induced apoptotic cell death by barley sprout extract (BSE) in HepG2 cells. Cells were pretreated with BSE (1 mg/mL) for 12 h before treatment with *t*-BHP (300 µM) for 18 h, followed by (**A**) FACS analysis of propidium iodide uptake and annexin V binding in non-permeabilized cells (lower-left, live cells; lower-right, early apoptotic cells; upper-right, late apoptotic cells); (**B**) quantification of apoptotic and (**C**) live cells based on 3 independent experiments. Values with different letters are significantly different by analysis of variance (ANOVA) followed by Newman–Keuls multiple comparisons test (*p* < 0.05).

To investigate the antioxidant effect of BSE, we examined its ROS scavenging ability and cell viability following *t*-BHP exposure in HepG2 cells. Pretreatment with BSE for 1 h significantly reduced *t*-BHP-induced ROS generation (Figure 4A) and cell death (Figure 4B) in a dose-dependent manner. Inhibition of *t*-BHP-induced oxidative cell death by BSE was confirmed by flow cytometry analysis using annexin V (Figure 5). Exposure to *t*-BHP induced significant apoptotic cell death, which was prevented by treatment with BSE (1 mg/mL).

### 3.4. Prevention of t-BHP-Induced Mitochondrial Dysfunction by BSE in HepG2 Cells

**Figure 6:**
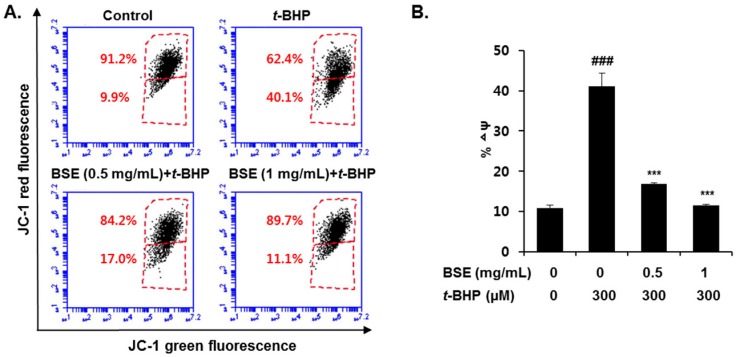
Prevention of *t*-BHP-induced mitochondrial dysfunction by barley sprout extract (BSE) in HepG2 cells. Cells were pretreated with BSE for 12 h before treatment with *t*-BHP (300 µM) for 18 h, followed by (**A**) FACS analysis after incubation with JC-1 (10 µM) and (**B**) quantification of mitochondrial membrane potential (∆ψ). Data are represented as the mean ± SD based on 3 independent experiments. ^###^ Significantly different from vehicle-treated cells, *p* < 0.001. *** Significantly different from *t*-BHP-treated cells, *p* < 0.001 (Student’s *t*-test).

Mitochondria are the major site of ROS generation, which induces oxidative stress under pathological conditions. To determine whether BSE prevents mitochondrial dysfunction through its ROS scavenging effect, we measured the ∆ψ using JC-1. It selectively enters the mitochondria and reversibly changes its color as the ∆ψ changes. As shown in Figure 6, *t*-BHP exposure in HepG2 cells significantly increased the P3 cell population, suggesting that the mitochondrial membranes were depolarized. However, pretreatment with BSE (0.5 and 1 mg/mL) restored the elevated ∆ψ to the control level. These results indicate that BSE effectively suppresses *t*-BHP-induced mitochondrial dysfunction and protects the HepG2 cells.

### 3.5. Induction of Nrf2 Activity in HepG2 Cells by BSE

**Figure 7:**
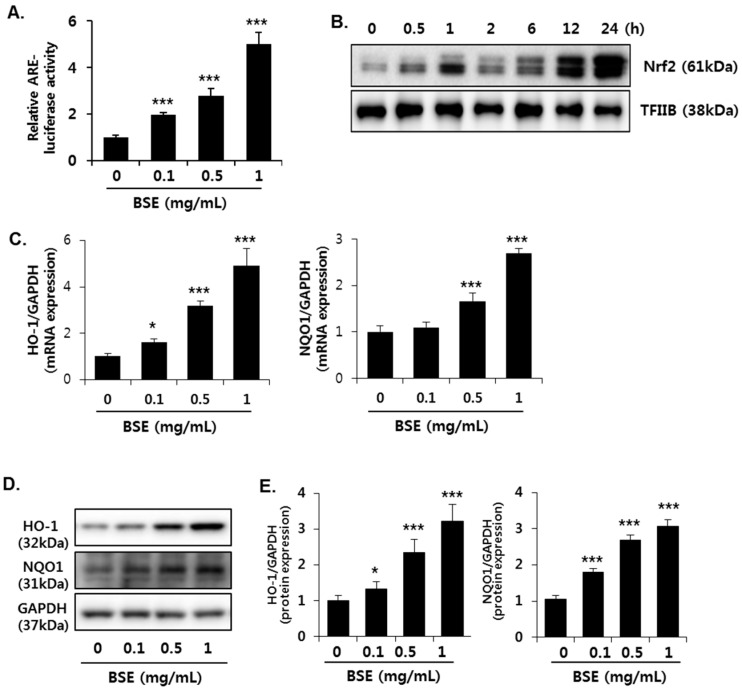
Induction of Nrf2 activity in HepG2 cells by barley sprout extract (BSE). (**A**) Cells were exposed to indicate concentrations of BSE for 12 h, and Nrf2 transactivation was determined by ARE-luciferase activity; (**B**) cells were exposed to BSE (1 mg/mL) for 24 h, and Nrf2 protein expression in the nucleus was determined by Western blotting. TFIIB was used as a loading control of nuclear fraction; cells were exposed to indicate concentrations of BSE for 18 h, followed by (**C**) determination of HO-1 and NQO-1 gene expression using real-time PCR; (**D**) visualization of HO-1 and NQO1 protein expression; and (**E**) quantification of their protein levels. Quantitative analysis of the target genes and blots normalized by GAPDH expression. Each value represents the mean ± SD. * and *** Significantly different from vehicle-treated control cells at *p* < 0.05 and < 0.001, respectively (Student’s *t*-test).

To determine whether Nrf2 is involved in the antioxidant effect of BSE, we measured ARE activation using reporter gene analysis. ARE luciferase constructs, containing three tandem repeats of ARE in the 5′-upstream region of NQO1, were transfected into HepG2 cells. Exposure to BSE significantly increased the luciferase activity of the NQO1-ARE reporter construct, suggesting that BSE induces transcriptional activation of Nrf2 (Figure 7A). BSE (1 mg/mL) increased the translocation of Nrf2 into the nucleus in a time-dependent manner till 24 h (Figure 7B). Nuclear translocation of activated Nrf2 is an important upstream regulatory step for the expression of its target genes, such as HO-1 and NQO1; therefore, we measured their levels of gene expression (Figure 7C) as well as protein (Figure 7D,E) in the cells. Consistent with the enhanced Nrf2 activity, BSE exposure dose-dependently increased the expression of HO-1 and NQO1 in both mRNA and protein levels.

### 3.6. Effect of BSE on the De Novo Synthesis of GSH

**Figure 8:**
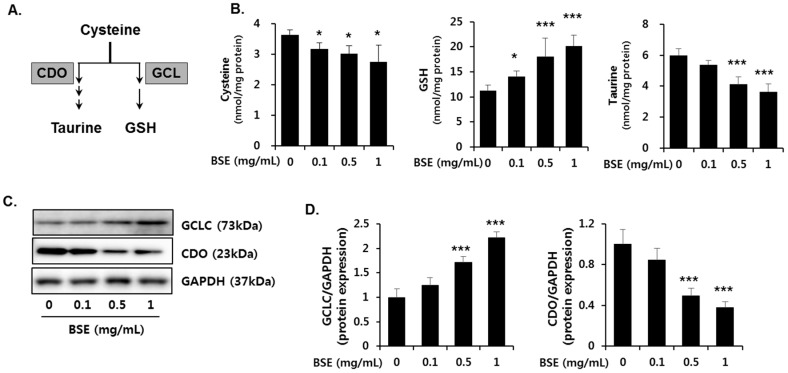
Effect of barley sprout extract (BSE) on the de novo synthesis of GSH and taurine in HepG2 cells. (**A**) Synthesis of taurine and GSH from cysteine in the liver. Cells were exposed to indicate concentrations of BSE for 18 h and (**B**) the cellular concentration of metabolites including cysteine, GSH, and taurine were determined using HPLC; (**C**) protein expression of GCLC, CDO, and GAPDH in the whole-cell lysate and (**D**) quantitative analysis of the blots normalized by GAPDH expression. Each value represents the mean ± SD. *, *** Significantly from vehicle-treated control cells at *p* < 0.05 and 0.001, respectively (Student’s *t*-test).

**Figure 9:**
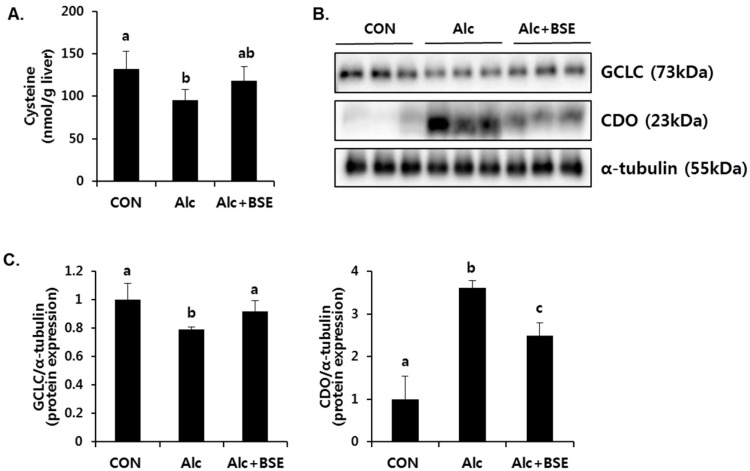
Effect of barley sprout extract (BSE) on the concentration of cysteine and the protein expression of GCLC, CDO in livers of alcohol-treated mice. (**A**) Cysteine concetration; (**B**) protein expression of GCLC, CDO, and α-tubulin in the liver and (**C**) quantitative analysis of the blots normalized by α-tubulin expression as a loading control. Each value represents mean ± SD of 6 mice. Values with different letters are significantly different by analysis of variance (ANOVA) followed by Newman–Keuls multiple comparisons test (*p* < 0.05). GCLC, glutamate-cysteine ligase catalytic subunit; CDO, cysteine dioxygenase; CON, control diet-fed mice; Alc, alcoholic diet-fed mice; Alc+BSE, alcoholic diet-fed mice treated with barley sprout extract (100 mg/kg).

GSH plays an important role in trapping reactive metabolites and is considered as the most potent endogenous antioxidant molecule. De novo synthesis of GSH depends on both the availability of cysteine and the level of glutamyl cysteine ligase (GCL) enzyme (Figure 8A). GCL is the rate-limiting enzyme in the de novo synthesis of GSH and is composed of a catalytic (GCLC) and a modifier (GCLM) subunit. GCLC gene expression, which is controlled by Nrf2, is critical in GSH synthesis. Therefore, we determined whether Nrf2 activation by BSE regulates the synthesis of GSH. BSE exposure in HepG2 cells dose-dependently increased the GSH concentration (Figure 8B) with an increased GCLC protein expression (Figure 8C,D). Further, it dose-dependently decreased the cysteine concentration (Figure 8B) and the protein expression of CDO (Figure 8C,D) that mediates the catabolism of cysteine to taurine. Accordingly, reduced cellular taurine levels were observed (Figure 8B). On the other hand, alcohol-treated mice enhanced CDO expression accompanied with decreased level of GCLC expression and cysteine in the liver, which was significantly inhibited by BSE supplementation (Figure 9). These results suggested that BSE enhances GSH synthesis by increasing the level of cysteine available for GSH synthesis and GCLC protein expression.

## 4. Discussion

We previously showed that BSE treatment significantly prevents liver injury induced by chronic alcohol administration in mice [23]. It inhibits hepatic lipid accumulation and reduces serum biochemical markers of liver injury, such as aspartate aminotransferase and alanine aminotransferase. Further, it prevents the depletion of hepatic GSH and suppresses the inflammatory responses, thereby attenuating alcohol-induced liver injury.

Orally administrated alcohol is biotransformed to acetaldehyde and oxygen free radicals, which are responsible for the formation of lipid peroxidation products in the liver [28,29]. Alcohol-induced lipid peroxidation induces early oxidative stress and leads to GSH depletion [30,31,32]. GSH or γ-l-glutamyl-l-cysteinylglycine, is a tripeptide that is ubiquitous in all mammalian tissues. It is the most abundant non-protein thiol that defends against oxidative stress. Therefore, it is reasonable to administer a GSH precursor or an exogenous antioxidant to reduce the oxidative stress-induced liver injury in alcohol-fed mice [33,34,35,36]. In the present study, we focused on the Nrf2-mediated antioxidant effect of BSE and increase in GSH synthesis to inhibit alcohol-induced oxidative liver injury.

Nrf2 plays a critical role in the progression of ALD and oxidative hepatotoxicity. Alcohol significantly increases liver failure-associated mortality in Nrf2-knockout mice, but not in wild-type mice [37]. Specifically, the loss of Nrf2 markedly increases lipid accumulation and significantly depletes GSH in the livers of alcohol-fed mice. In addition, alcohol intake exacerbates the hepatic inflammatory response by activating the interleukin-6/Stat-3 pathway in Nrf2-knockout mice. However, constitutively activated Nrf2 by gene manipulation prevents oxidative stress and lipid accumulation in the liver of alcohol-fed mice by increasing the expression of antioxidant genes and decreasing the expression of lipogenic genes [38]. Treatment with an Nrf2 inducer is proven to ameliorate alcohol-induced liver injury. Curcumin, isolated from the rhizomes of *Curcuma longa*, is a potent Nrf2 inducer that alleviates alcohol-induced lipid accumulation in the rat liver and human hepatocytes by regulating lipid metabolism [39]. Further, curcumin inhibits alcohol-induced necroptosis in hepatocytes through the Nrf2/p53 pathway [20].

In the present study, BSE exposure significantly increased ARE-luciferase activity, nuclear Nrf2 level, and expression of its target genes, such as NQO1, HO-1, and GCLC, suggesting transcriptional activation of Nrf2 by BSE. Subsequently, the increased GCLC protein expression resulted in enhanced biosynthesis of GSH to prevent oxidative stress. In particular, this study predominantly focused on the increase in GSH synthesis by BSE. Biosynthesis of GSH is regulated by the availability of cysteine (substrate) and GCLC (the rate-limiting enzyme in the synthetic pathway) [40]. Cysteine is used to form taurine as well as GSH, and its availability is a major determinant for partitioning of the cysteine sulfur to GSH or taurine [41]. It was shown that cysteine concentration directly affects CDO protein level by changes in the rate of CDO ubiquitination and proteasomal degradation [42]. Therefore, a high concentration of cysteine induces the conversion of more cysteine to taurine through up-regulated CDO expression, whereas GCLC is induced when cysteine availability is low, ensuring that more cysteine is conserved as GSH [41]. In this study, the reduced expression of CDO (the rate-limiting enzyme in taurine synthesis) accompanied with decreased cysteine level was inversely related to the increased expression of GCLC by BSE, thereby suggesting that cysteine was utilized to synthesize GSH. In conclusion, our results show that BSE induces Nrf2 activity, resulting in maintenance of hepatic GSH and preventing alcohol-induced oxidative liver injury.

## Figures and Tables

**Table 1:** List of mouse primer used for real-time reverse transcription-polymerase chain reaction (RT–PCR).

Genes	Primer Sequences	
HO-1	F: CGGGCCAGCAACAAAGTG	R: AGTGTAAGGACCCATCGGAGAA
NQO1	F: AGGCTGGTTTGAGCGAGT	R: ATTGAATTCGGGCGTCTGCTG
GAPDH	F: ATCACCATCTTCCAGGAGCGA	R: GCCAGTGAGCTTCCCGTTCA
